# Reduced residual cholesterol is associated with increased mortality risk in patients with aneurysmal subarachnoid Hemorrhage: a retrospective study

**DOI:** 10.3389/fneur.2025.1595387

**Published:** 2025-09-09

**Authors:** Xihang Zeng, Ruoran Wang, Jianguo Xu

**Affiliations:** Department of Neurosurgery, West China Hospital, Sichuan University, Chengdu, China

**Keywords:** residual cholesterol, lipid profile, subarachnoid hemorrhage, intracranial aneurysm, mortality

## Abstract

**Background:**

Residual cholesterol has been confirmed to be associated with the incidence of stroke and its prognosis. However, there is no study exploring the relationship between residual cholesterol and mortality in cases of aneurysmal subarachnoid hemorrhage (aSAH). Therefore, this study investigated the association between residual cholesterol levels and aSAH mortality.

**Methods:**

A restricted cubic spline was used to show the relationship between residual cholesterol and mortality risk associated with aSAH. Univariate and multivariate logistic regression models were employed to identify independent risk factors for mortality. The independent risk factors identified in the multivariate logistic regression were combined to develop a predictive model for mortality risk. The receiver operating characteristic curve (ROC) was used to evaluate the predictive value of residual cholesterol and the developed predictive model.

**Results:**

Among the aSAH patients included in the study, 20.0% experienced 30-day mortality. There were no significant differences in total cholesterol (*p* = 0.121), low-density lipoprotein cholesterol (*p* = 0.143), and triglycerides (*p* = 0.254) between survivors and non-survivors; however, high-density lipoprotein cholesterol (*p* = 0.021) was higher in non-survivors. Residual cholesterol (*p* < 0.001) was significantly lower among non-survivors. Multivariate logistic regression analysis revealed seven significant risk factors related to the mortality of aSAH including the Glasgow Coma Scale (GCS) (*p* < 0.001), modified Fisher Scale (mFisher) (*p* = 0.032), white blood cell count (*p* = 0.004), glucose levels (*p* = 0.008), residual cholesterol (*p* = 0.047), delayed cerebral ischemia (*p* < 0.001), and surgical options (*p* < 0.001). A predictive model for aSAH mortality was developed by combining these seven significant factors. The area under the ROC (AUC) for this predictive model was 0.911, while the AUC for residual cholesterol was 0.603.

**Conclusion:**

Residual cholesterol is negatively associated with mortality risk in aSAH. Evaluating residual cholesterol is helpful in risk stratification of aSAH patients.

## 1 Introduction

Aneurysmal subarachnoid hemorrhage (aSAH) has an annual incidence of 9.1 cases per 100,000 individuals and is associated with a poor prognosis, with a mortality rate as high as 66.7% (1, 2). Risk stratification of aSAH severity at the early stage is beneficial for physicians, allowing them to adopt personalized treatments and improve the prognosis of aSAH. Some clinical scoring systems have been developed to assess the severity of aSAH, including the World Federation of Neurosurgical Society (WFNS) scale, the Hunt-Hess scale, and the modified Fisher scale (mFisher). Additionally, many biomarkers have been explored to predict the prognosis of aSAH, such as glial fibrillary acidic protein, copeptin, melatonin, interleukin-6, interleukin-33, nicotinamide adenine dinucleotide phosphate oxidase 4, cellular prion protein, and growth-arrest-specific protein 6 (3–10). These uncommon biomarkers are rarely measured in clinical settings, leading to higher medical costs. Attracting much attention from researchers, biochemical markers derived from routine blood examinations, such as the neutrophil-to-lymphocyte ratio, the neutrophil-to-albumin ratio, and the white blood cell-to-hemoglobin ratio, are widely explored due to low cost and practical convenience (11–13).

The lipid profile, a key component of blood biochemistry examinations, has garnered considerable interest from researchers because of its significant role in the development and progression of cardiovascular and cerebrovascular diseases (14–17). In addition to the traditional lipid profile, which includes total cholesterol, low-density lipoprotein cholesterol (LDL-C), high-density lipoprotein cholesterol (HDL-C), and triglycerides, some derived indicators have been developed, including residual cholesterol, calculated as total cholesterol minus the sum of HDL-C and LDL-C (18). Residual cholesterol has been confirmed to be related to the incidence and prognosis of ischemic stroke (19–21). There was no study exploring the association between residual cholesterol and the prognosis of aSAH. This study aimed to validate the correlation between mortality associated with aSAH and residual cholesterol levels.

## 2 Methods

### 2.1 Participants

aSAH patients who were treated in the West China Hospital (from January 2017 to June 2019) were enrolled in this retrospective study. The diagnosis of aSAH was confirmed through radiological signs observed on digital subtraction angiography (DSA) or computed tomography angiography (CTA). Some patients were excluded from the study based on specific criteria: (1) admission to our medical center 24 h after initial aneurysm rupture-related symptoms, (2) transfer from other hospitals, (3) incomplete records of the included variables, and (4) history of other neurological diseases such as stroke and central nervous system tumors. A total of 695 aSAH patients were finally enrolled in the study. The Ethics Committee of West China Hospital (2021–1,684) approved this retrospective study. Informed consent was obtained from patients or their legal representatives upon admission to our hospital.

### 2.2 Variables

Age, gender, smoking habit, alcohol consumption, and comorbidities, including diabetes mellitus and hypertension, were recorded. Systolic blood pressure and diastolic blood pressure were collected at admission. Clinical scoring systems, including the Glasgow Coma Scale (GCS) and the modified Fisher Scale (mFisher) scores at admission, were selected. Aneurysm-related information, including aneurysm location and multiple aneurysms, was identified based on radiological findings. Laboratory tests were collected within 4 h after admission, including white blood cell count, hemoglobin levels, platelets, triglycerides, total cholesterol, HDL-C, LDL-C, glucose levels, and lactate dehydrogenase. Residual cholesterol was calculated as total cholesterol-(HDL-C + LDL-C). Delayed cerebral ischemia was confirmed based on any one of the following two standards: (1) a dramatic decrease in the GCS score of ≥2 points (decrease in the total score or one component of the GCS, excluding other causes identified through clinical assessments and cerebral imaging) and (2) focal neurological impairment. Surgical options were classified into three categories: clipping, coiling, and no surgery, were classified. The primary outcome was set as 30-day mortality.

### 2.3 Statistical method

The normality of the variables was tested using the Kolmogorov–Smirnov test. Overall, patients were categorized into two groups: non-survivors and survivors. The difference in variables with normal distribution (expressed as mean ± standard deviation) and variables with non-normal distribution [expressed as median (interquartile range)] between non-survivors and survivors was verified utilizing the Student’s t-test and Mann–Whitney *U*-test, respectively. The difference in categorical variables [expressed as number (percentage)] was tested utilizing the chi-squared test. Spearman’s correlation analysis was performed to explore the relationship between residual cholesterol and other variables. A restricted cubic spline (RCS) was plotted to visually show the dose-dependent correlation between residual cholesterol and the mortality risk of aSAH. Significant risk factors for aSAH mortality identified through univariate logistic regression were validated in the multivariate logistic regression. The independent risk factors in the multivariate logistic regression were combined to develop a predictive model for mortality. Subgroup analyses were performed to evaluate the relationship between residual cholesterol levels and mortality risk, with additional testing for potential interaction effects across clinically relevant stratification variables. The receiver operating characteristic curve (ROC) was plotted to assess the predictive accuracy of residual cholesterol and the developed predictive model. A nomogram and calibration plots of the predictive model were plotted. All these analyses were conducted using R (version 4.3.2).

## 3 Results

### 3.1 Characteristics of enrolled aSAH patients

Among the aSAH patients included in the study, 20.0% experienced 30-day mortality (Table 1). Non-survivors were older (*p* = 0.012) with lower GCS (*p* < 0.001) and higher mFisher (*p* < 0.001) scores. No significant differences were observed between survivors and non-survivors in terms of gender distribution (*p* = 0.719), prevalence of smoking (*p* = 0.312), alcohol consumption (*p* = 0.518), or comorbidities including diabetes mellitus (*p* = 0.429) and hypertension (*p* = 0.116). Laboratory tests revealed that white blood cell count (*p* < 0.001), glucose levels (*p* < 0.001), and lactate dehydrogenase levels (*p* < 0.001) were higher among non-survivors. Total cholesterol (*p* = 0.121), low-density lipoprotein cholesterol (*p* = 0.143), and triglycerides (*p* = 0.254) were not different between survivors and non-survivors, while high-density lipoprotein cholesterol (*p* = 0.021) was higher among non-survivors. Residual cholesterol (*p* < 0.001) was significantly lower among non-survivors. Non-survivors were more likely to receive no surgical intervention for the ruptured aneurysm (*p* < 0.001). Finally, non-survivors had a shorter hospital stay (*p* < 0.001) but no difference in ICU stay length (*p* = 0.191) compared to survivors.

**Table 1:** Baselines of aSAH patients.

	Overall (*n* = 695)	Survivors (*n* = 556, 80.0%)	Non-survivors (*n* = 139, 20.0%)	*p*
Age (years)	55 (49–65)	55 (48–64)	59 (50–69)	0.012
Male gender (*n*, %)	239 (34.4%)	193 (34.7%)	46 (33.1%)	0.719
Smoking (*n*, %)	104 (15.0%)	87 (15.6%)	17 (12.2%)	0.312
Alcohol (*n*, %)	123 (17.7%)	101 (18.2%)	22 (15.8%)	0.518
Diabetes mellitus (*n*, %)	34 (4.9%)	29 (5.2%)	5 (3.6%)	0.429
Hypertension (*n*, %)	296 (42.6%)	245 (44.1%)	51 (36.7%)	0.116
Systolic blood pressure (mmHg)	146 (128–166)	147 (129–166)	141 (126–168)	0.453
Diastolic blood pressure (mmHg)	85 (76–95)	85 (76–95)	86 (77–95)	0.374
GCS	13 (11–14)	14 (12–15)	7 (6–12)	<0.001
mFisher	4 (2–4)	3 (2–4)	4 (3–4)	<0.001
Multiple aneurysm (*n*, %)	66 (9.5%)	49 (8.8%)	17 (12.2%)	0.219
Laboratory tests				
White blood cell (10^9^/L)	10.32 (8.01–13.52)	9.97 (7.75–12.53)	12.86 (9.21–17.25)	<0.001
Platelet (10^9^/L)	165 (128–207)	166 (130–204)	162 (122–214)	0.825
Hemoglobin (g/L)	125 (110–136)	125 (109–135)	126 (112–139)	0.214
Glucose (mmol/L)	6.60 (5.58–8.11)	6.30 (5.41–7.56)	7.67 (6.52–10.47)	<0.001
Lactate dehydrogenase (U/L)	195 (165–241)	189 (160–226)	232 (190–294)	<0.001
Total cholesterol (mmol/L)	4.20 ± 1.03	4.23 ± 1.01	4.08 ± 1.10	0.121
High density lipoprotein (mmol/L)	1.24 (0.95–1.53)	1.23 (0.94–1.52)	1.33 (1.05–1.56)	0.021
Low density lipoprotein (mmol/L)	2.40 (1.86–2.97)	2.42 (1.89–2.98)	2.31 (1.72–2.92)	0.143
Triglyceride (mmol/L)	1.07 (0.79–1.47)	1.08 (0.80–1.49)	1.04 (0.74–1.38)	0.254
Residual cholesterol (mmol/L)	0.44 (0.27–0.64)	0.46 (0.29–0.66)	0.36 (0.18–0.56)	<0.001
Aneurysm location (*n*, %)				
Anterior circulation	639 (92.0%)	518 (93.2%)	121 (87.1%)	0.018
Posterior circulation	56 (8.1%)	38 (6.8%)	18 (13.0%)	
Delayed cerebral ischemia (*n*, %)	116 (16.7%)	70 (12.6%)	46 (33.1%)	<0.001
Surgical options (*n*, %)				
None	111 (16.0%)	41 (7.4%)	70 (50.4%)	<0.001
Clipping	513 (73.8%)	457 (82.2%)	56 (40.3%)	
Coiling	71 (10.2%)	58 (10.4%)	13 (9.4%)	
Length of ICU stay (days)	3 (0–9)	3 (0–9)	4 (0–10)	0.191
Length of hospital stay (days)	12 (9–19)	13 (10–19)	7 (3–13)	<0.001

GCS, Glasgow Coma Scale; mFisher, modified Fisher.

### 3.2 Correlation between residual cholesterol and aSAH mortality

**Figure 1:**
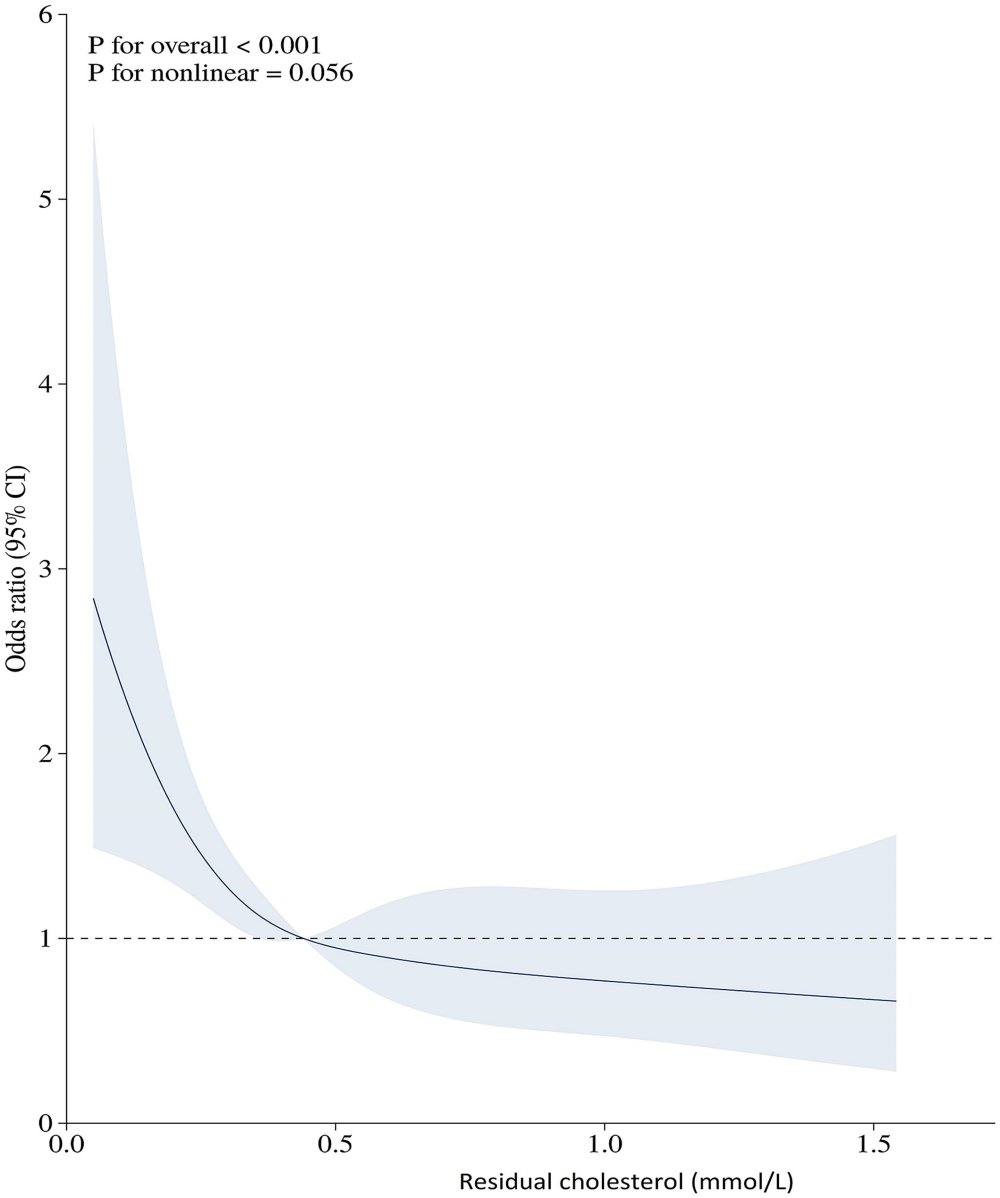
Relationship between residual cholesterol and aSAH mortality is shown by the restricted cubic spline.

**Figure 2:**
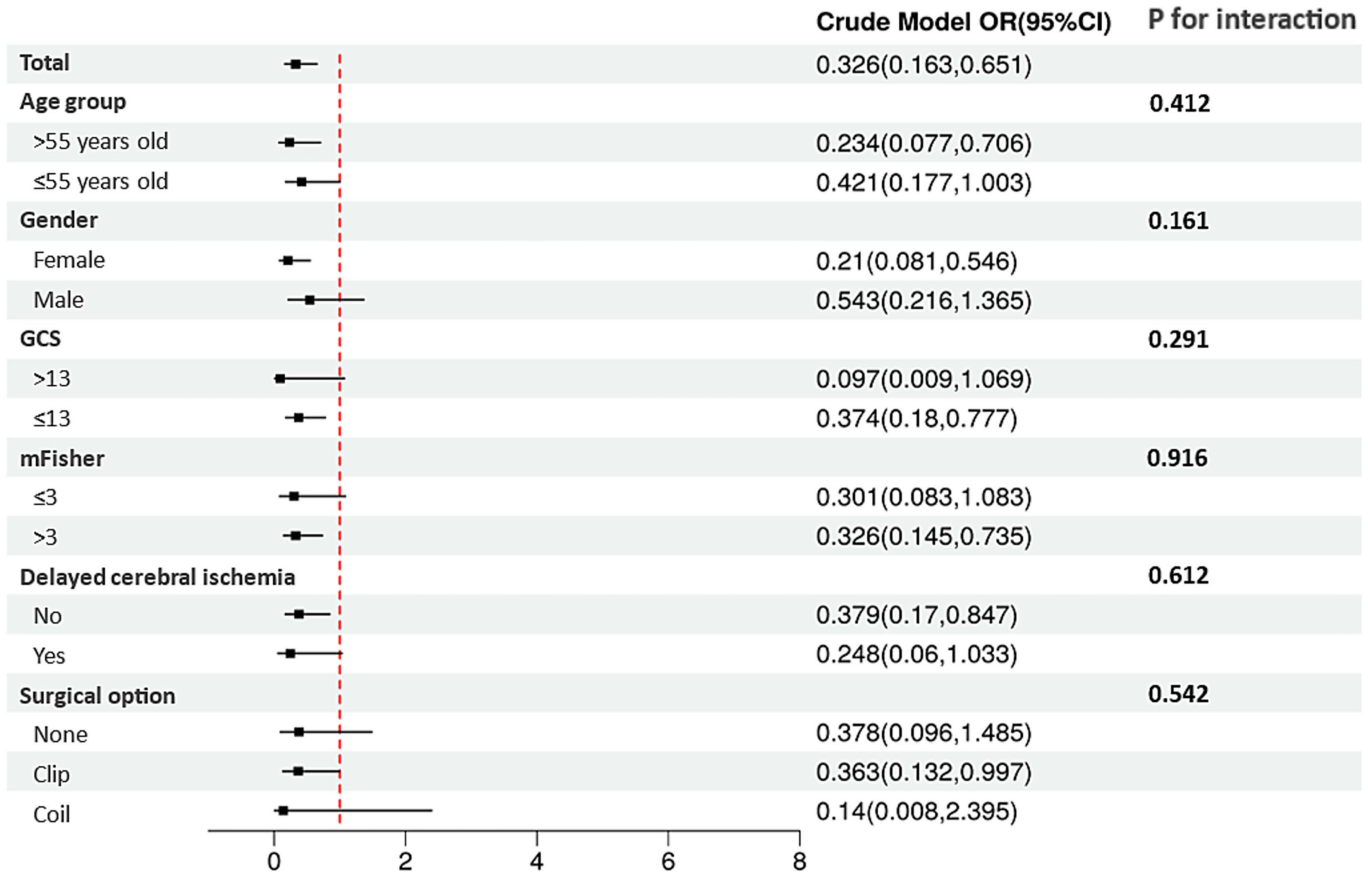
Forest plot of Multiple logistic regression.

Spearman’s correlation analysis revealed no significant association between residual cholesterol and either GCS score (*p* = 0.084, *p* = 0.027) or mFisher grade (*p* = 0.003, *p* = 0.935) (Table 2). RCS analysis showed linear and negative correlations between residual cholesterol and aSAH mortality (*p* < 0.001) (Figure 1). A total of 11 potential risk factors for aSAH mortality were identified through univariate logistic regression, including age (*p* = 0.005), GCS score (*p* < 0.001), mFisher grade (*p* < 0.001), white blood cell count (*p* < 0.001), glucose levels (*p* < 0.001), lactate dehydrogenase (*p* < 0.001), high density lipoprotein (*p* = 0.048), residual cholesterol (*p* = 0.002), posterior circulation (*p* = 0.020), delayed cerebral ischemia (p < 0.001), and surgical options (*p* < 0.001) (Table 3). Multiple logistic regression identified seven independent significant risk factors for aSAH mortality, including GCS score (*p* < 0.001), white blood cell count (*p* = 0.004), mFisher grade (*p* = 0.032), glucose levels (*p* = 0.008), residual cholesterol (*p* = 0.047), surgical options (*p* < 0.001), and delayed cerebral ischemia (*p* < 0.001). Although subgroup analyses revealed varying *p*-values across different strata, no statistically significant interaction effects were observed between residual cholesterol and age, gender, GCS score, mFisher grade, delayed cerebral ischemia, or surgical treatment options (all *p*-interaction > 0.05). The absence of detectable interaction effects may represent a type II error secondary to a limited sample size (Table 4; Figure 2).

**Table 2:** Spearman correlation analysis between residual cholesterol and severity scores of aSAH patients.

	*R* coefficient	*p*
GCS	0.084	0.027
mFisher	0.003	0.935
Total cholesterol (mmol/L)	0.097	0.010
High density lipoprotein	−0.557	<0.001
Low density lipoprotein	0.067	0.080
Triglyceride	0.725	<0.001

OR, odds ratio; CI, confidence interval; GCS, Glasgow Coma Scale; mFisher, modified Fisher.

**Table 3:** Risk factors for the mortality of aSAH analyzed by the logistic regression.

Variables	Univariate analysis			Multivariate analysis		
	OR	95% CI	*p*	OR	95% CI	*p*
Age	1.024	1.007–1.040	0.005	1.009	0.987–1.032	0.422
Male gender	0.930	0.627–1.380	0.719			
Smoking	0.847	0.512–1.402	0.519			
Alcohol	0.751	0.431–1.311	0.314			
Diabetes mellitus	0.678	0.258–1.785	0.431			
Hypertension	0.736	0.501–1.079	0.117			
Systolic blood pressure	0.998	0.991–1.006	0.635			
Diastolic blood pressure	1.007	0.994–1.019	0.298			
GCS	0.677	0.634–0.723	<0.001	0.750	0.689–0.814	<0.001
mFisher	1.609	1.299–1.993	<0.001	1.394	1.036–1.903	0.032
Multiple aneurysm	1.442	0.802–2.591	0.221			
White blood cell	1.165	1.117–1.215	<0.001	1.088	1.027–1.154	0.004
Platelet	1.000	0.997–1.003	0.872			
Hemoglobin	1.004	0.996–1.013	0.322			
Glucose	1.289	1.203–1.382	<0.001	1.140	1.035–1.257	0.008
Lactate dehydrogenase	1.006	1.003–1.008	<0.001	1.002	0.999–1.004	0.116
Total cholesterol	0.866	0.721–1.039	0.122			
High density lipoprotein	1.516	1.004–2.291	0.048	0.853	0.413–1.733	0.663
Low density lipoprotein	0.838	0.668–1.050	0.124			
Triglyceride	1.005	0.864–1.170	0.948			
Residual cholesterol	0.326	0.163–0.651	0.002	0.350	0.118–0.925	0.047
Aneurysm location						
Anterior circulation	1.000	Reference		1.000	Reference	
Posterior circulation	2.028	1.119–3.675	0.020	0.975	0.392–2.399	0.955
Delayed cerebral ischemia	3.434	2.227–5.296	<0.001	4.991	2.747–9.187	<0.001
Surgical options						
None	1.000	Reference		1.000	Reference	
Clipping	0.072	0.045–0.115	<0.001	0.08	0.042–0.149	<0.001
Coiling	0.131	0.064–0.268	<0.001	0.129	0.048–0.323	<0.001

**Table 4:** Subgroup analysis for the association between residual cholesterol and the mortality of aSAH patients.

	OR	95% CI	*p*	*p* for interaction
Total	0.326	0.163–0.651	0.002	
Age				0.412
>55	0.234	0.077–0.706	0.010	
≤55	0.421	0.177–1.003	0.051	
Gender				0.161
Female	0.210	0.081–0.546	0.001	
Male	0.543	0.216–1.365	0.194	
GCS				0.291
>13	0.097	0.009–1.069	0.057	
≤13	0.374	0.180–0.777	0.008	
mFisher				0.916
≤3	0.301	0.083–1.083	0.066	
>3	0.326	0.145–0.735	0.007	
Delayed cerebral ischemia				0.612
No	0.379	0.170–0.847	0.018	
Yes	0.248	0.060–1.033	0.055	
Surgical options				0.542
None	0.378	0.096–1.485	0.164	
Clip	0.363	0.132–0.997	0.049	
Coil	0.140	0.008–2.395	0.175	

OR, odds ratio; CI, confidence interval; GCS, Glasgow Coma Scale; mFisher, modified Fisher.

### 3.3 Prognostic value of residual cholesterol among aSAH

**Figure 3:**
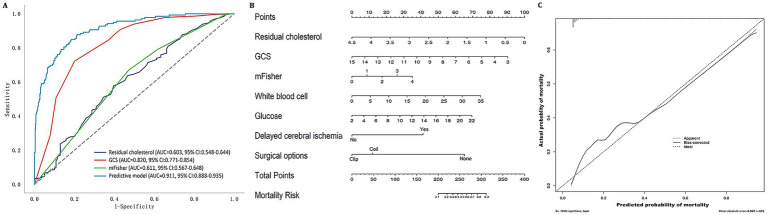
**(A)** Receiver operating characteristic curve of residual cholesterol for predicting mortality in aSAH patients. **(B)** Nomogram of the predictive model for mortality in aSAH patients. **(C)** Calibration plot of the predictive model for mortality in aSAH patients. GCS, Glasgow Coma Scale; mFisher, modified Fisher; AUC, area under the receiver operating characteristic curve.

Seven significant factors from multivariate logistic regression were combined to develop the predictive model for aSAH mortality (Table 5). The predictive accuracy of residual cholesterol (AUC = 0.603) and the predictive model (AUC = 0.911) was evaluated using ROC analysis (Table 6; Figure 3A). The nomogram and calibration curve of the predictive model are presented in Figures 3B,C.

**Table 5:** The logistic predictive model for the mortality of aSAH patients.

	OR	95% CI	*p*
Residual cholesterol	0.417	0.171–0.927	0.044
GCS	0.744	0.683–0.806	<0.001
mFisher	1.411	1.050–1.924	0.025
White blood cell	1.088	1.030–1.15	0.003
Glucose	1.147	1.042–1.261	0.005
Delayed cerebral ischemia	5.063	2.808–9.237	<0.001
Surgical options			
None	1.000	Reference	
Clip	0.077	0.041–0.14	<0.001
Coil	0.123	0.048–0.297	<0.001

OR, odds ratio; CI, confidence interval; GCS, Glasgow Coma Scale; mFisher, modified Fisher.

**Table 6:** The predictive value of residual cholesterol and the logistic model for the mortality of aSAH patients.

	AUC (95% CI)	Sensitivity	Specificity	Youden index	Cut off value
Residual cholesterol	0.603 (0.548–0.644)	0.577	0.604	0.182	0.42
GCS	0.820 (0.771–0.854)	0.723	0.799	0.522	13
mFisher	0.611 (0.567–0.648)	0.669	0.529	0.198	4
Predictive model	0.911 (0.888–0.935)	0.849	0.817	0.665	0.168

AUC, area under the receiver operating characteristic curve; CI, confidence interval; GCS, Glasgow Coma Scale; mFisher, modified Fisher.

## 4 Discussion

The lipid profile from this study showed that total cholesterol, LDL-C, and triglycerides were relatively lower in non-survivors of aSAH, although this difference was not statistically significant. Similarly, one study found that aSAH patients with unfavorable outcomes had lower levels of total cholesterol (22). This fact means that the cholesterol levels are a protective factor for the prognosis of aSAH. However, one previous study confirmed that higher levels of serum triglycerides and cholesterol were related to fewer occurrences of SAH (23). The protective effect of cholesterol and triglycerides on the outcome of aSAH has not been verified. HDL-C was higher among non-survivors of aSAH, indicating that high HDL-C may be a risk factor for aSAH mortality. One previous study showed that both low total cholesterol and low HDL-C were independent predictors of increased mortality and delayed cerebral ischemia in aSAH patients (24). Only 75 aSAH patients were included in the study. Additionally, HDL-C was not found to be significantly related to mortality in our multivariate logistic regression. The prognostic effect of HDL-C among aSAH patients deserves further exploration. Nevertheless, only residual cholesterol was finally identified as an independent factor for aSAH mortality in our multivariate logistic regression. Residual cholesterol was significantly higher in survivors of aSAH, indicating that increased levels of residual cholesterol were a protective factor for the prognosis of aSAH.

Residual cholesterol has been confirmed to be related to the incidence and prognosis of ischemic stroke (19, 20). One study showed that higher levels of residual cholesterol were related to an increased risk of ischemic stroke but not hemorrhagic stroke (19). Other studies also found that elevated levels of residual cholesterol were a risk factor for acute ischemic stroke (19, 25–27). Positive effects of elevated levels of residual cholesterol on outcomes for acute ischemic stroke patients were also confirmed (28, 29). In contrast, one study showed that increased levels of residual cholesterol were negatively related to parenchymal hemorrhage after endovascular treatment for acute ischemic stroke of the anterior circulation (30). Another study showed that a low residual cholesterol level was associated with bleeding events during the acute stage of ischemic stroke or transient ischemic attack (20). It appears that increased levels of residual cholesterol were related to a lower risk of hemorrhagic events. Similarly, our findings showed that higher levels of residual cholesterol were a protective factor for the prognosis of aSAH, which is a common type of hemorrhagic stroke.

Residual cholesterol primarily consists of the cholesterol found in intermediate-density lipoprotein, very-low-density lipoprotein, triglyceride-rich lipoproteins, and chylomicron remnants (31). High residual cholesterol levels are associated with persistent arterial wall inflammation after endothelial injury, leading to hyperproliferation of vascular smooth muscle cells and neointimal hyperplasia (32–34). Although residual cholesterol has been confirmed to promote atherosclerosis, the mechanisms behind the inverse relationship between residual cholesterol and the mortality risk of aSAH warrant exploration. Some potential mechanisms need further experimental verification. First, cholesterol is known to play a crucial role in maintaining the integrity of cerebral small blood vessels and the blood–brain barrier (35, 36). Low levels of cholesterol could cause increased permeability and leakage of these vessels (37). Research has confirmed that elevated blood–brain barrier permeability contributed to delayed cerebral ischemia pathogenesis and poorer neurological outcomes after aSAH (38, 39). Additionally, serum cholesterol could affect platelet aggregation and, therefore, may promote bleeding events (40). Bleeding volume was an independent risk factor for the poor prognosis of aSAH patients (41).

Several limitations of this retrospective study need to be mentioned. First, this observational study enrolled aSAH patients from a single center in Southwest China. Some ineligible patients were excluded from the study. As a result, selection bias may have occurred, and the findings should be verified in other medical centers with a more generalized population affected by aSAH. Second, residual cholesterol levels may be influenced by various comorbidities, such as hyperlipidemia, atherosclerosis, and drugs that affect blood lipid levels. The findings of our study should be verified in future research, adjusting for the confounding effects of these factors. Third, markers of endothelial injury, inflammation, oxidation, and atherosclerosis were not measured. Therefore, we were unable to confirm the correlations between residual cholesterol and these pathophysiological processes to discover the underlying mechanisms. Fourth, only the initial residual cholesterol levels was collected, and the subsequent levels were not measured. Changes in residual cholesterol during hospitalization may provide additional prognostic value for aSAH patients.

## 5 Conclusion

Higher levels of residual cholesterol is related to lower mortality risk of aSAH. Evaluating residual cholesterol is useful in the risk stratification of aSAH patients.

## Data Availability

The raw data supporting the conclusions of this article will be made available by the authors, without undue reservation.
